# Fecal Immunoglobulin Levels as a Modifier of the Gut Microbiome in Patients with Common Variable Immunodeficiency

**DOI:** 10.1007/s10875-023-01469-9

**Published:** 2023-03-24

**Authors:** Christina Nöltner, Alla Bulashevska, Katrin Hübscher, Hanna Haberstroh, Bodo Grimbacher, Michele Proietti

**Affiliations:** 1 Institute for Immunodeficiency, Center for Chronic Immunodeficiency, Medical Center, Faculty of Medicine, Albert-Ludwigs-University of Freiburg, Breisacher Str. 115, 79106 Freiburg, Germany; 2 DZIF- German Center for Infection Research, Satellite Center Freiburg, Freiburg, Germany; 3 CIBSS- Centre for Integrative Biological Signalling Studies, Albert-Ludwigs-University, Freiburg, Germany; 4 Cluster of Excellence RESIST (EXC 2155), Hannover Medical School, Hannover, Germany; 5 Institute of Immunity and Transplantation, Royal Free Hospital, University College London, London, UK; 6 Department of Rheumatology and Immunology, Hannover Medical School, Hannover, Germany

**Keywords:** CVID, Intestinal microbiota, Fecal immunoglobulins, Alpha diversity

## Abstract

**Objective:**

Common variable immunodeficiency (CVID) is the most common clinically relevant entity of inborn errors of immunity. In these patients, an altered gut microbiome composition with reduced diversity has been described. We sought to investigate the fecal immunoglobulin levels and their impact on the gut microflora in patients with CVID.

**Methods:**

We analyzed the gut microbiome of 28 CVID patients and 42 healthy donors (HDs), including 21 healthy household controls, by sequencing the V3 and V4 regions of the bacterial 16S rRNA gene extracted from stool samples. The fecal levels of immunoglobulin A, M, and G of 27 CVID patients and 41 HDs were measured in the supernatant by ELISA and normalized for protein concentration.

**Results:**

We measured decreased IgA and increased IgG in stool samples from CVID patients compared to HDs. Decreased levels of fecal IgA and IgM were associated with reduced microbial diversity and increased dysbiosis. We identified a large number of significantly differentially abundant taxa, especially in patients with decreased IgA levels, but also in patients with decreased IgM levels compared to their counterparts.

**Conclusions:**

CVID patients have an altered gut microbiota composition, which is most prevalent in patients with decreased fecal IgA and IgM levels. In this study, we identify fecal immunoglobulins as a potential modifier of the gut microbiome in CVID patients.

**Supplementary Information:**

The online version contains supplementary material available at 10.1007/s10875-023-01469-9.

## Introduction

Common variable immunodeficiency (CVID) is the most prevalent clinically relevant inborn error of immunity with an estimated prevalence of 1 to 25,000 [1]. CVID is characterized by hypogammaglobulinemia of immunoglobulin G (IgG) and A (IgA), poor or absent specific antibody production to immunization, at an age greater than 4 years [2]. Patients suffer from recurrent infections, especially of the respiratory tract. About 70% of patients additionally develop non-infectious complications such as lymphoproliferation, organ-specific inflammation, and autoimmunity [3–5]. Their occurrence varies strongly, but causes significant morbidity and mortality [6]. While infections can be prevented by immunoglobulin replacement therapy and prophylactic antibiotics, the treatment of non-infectious complications remains a challenge [7].

In 2016, Jørgensen et al. were the first to describe a decreased microbial alpha diversity and a dysbiosis in stool samples from CVID patients. Reduced microbial diversity was most prevalent in patients with immune dysregulation, which correlated with low serum IgA levels [8]. Subsequent studies have confirmed an intestinal microbial dysbiosis in CVID patients [9–11]. Environmental factors seem to have a significant influence on microbial diversity, as shown by Fiedorova et al. who compared the microbiota between CVID patients and healthy controls from the same household [11]. Ho et al. detected increased levels of 16S rDNA of gut commensal bacteria in the serum of CVID patients, suggesting an increased microbial translocation [12].

Secretory immunoglobulins possess important functions at the mucosal surface. On the intestinal mucosa, IgA is the main isotype of secretory antibodies followed by IgM and IgG [13]. Multimeric IgA and IgM are produced locally in the mucosa by plasma cells, which are stimulated by the gut microbiota [14, 15].

By binding bacteria and activating the complement system, mucosal immunoglobulins preserve the microbial richness and stimulate the host’s immune response against bacterial infections [16–20]*.* Although present at comparatively low levels, secretory IgG can also be induced by the gut microbiota and coat bacteria, which prevents infection with *E. coli* and *Salmonella* [21].

While serum immunoglobulin levels are well-studied in CVID, very little is known about fecal immunoglobulin levels. In 2018, Shulzenko et al. detected fewer transcripts of genes indicative of IgA and IgG in duodenal biopsies in CVID patients compared to HDs [22].

The aim of this study was to investigate a possible link between fecal immunoglobulins and the gut microbiota composition in CVID by analysis of stool samples.

## Methods

### Sample Collection

Stool samples were collected from CVID patients and healthy donors (HDs), if available, from a healthy household donor (HHD). The collection period was between September 2015 and June 2017 at the Freiburg University Medical Center after ethical approval by the local ethics committee of the University of Freiburg (protocol no. 526/14). The study participants received stool tubes containing 8 ml of a stabilizing liquid (Stratec) and were obliged to send it within 24 h of sampling. Along with the sample, all study participants provided a questionnaire (based on the Kieler Fragebogen für Erwachsene, Studie zur Rolle des Mikrobioms, Version 1.2 of 01.08.2014), containing 86 questions about diet, environment, drug intake, and clinical symptoms.

### Exclusion Criteria

As exclusion criteria, the intake of antibiotics, probiotics, and immunosuppressive medication (except 5 mg prednisolone daily or less) within the last 4 weeks before sampling were chosen. Samples for which the questionnaire was inconclusive with regard to these criteria were also excluded. Samples from HHDs of CVID patients whose samples were excluded based on the exclusion criteria were included in the total pool of healthy donors (HDs). Collectively, we further analyzed stool samples of 28 CVID patients and 42 HDs, including 21 samples from corresponding HHDs.

### Fecal Microbial DNA Isolation

Upon arrival, stool samples were homogenized, divided into aliquots and stored at − 80 °C. Microbial DNA was extracted from a 2 ml aliquot using the QIAamp DNA Stool Mini Kit (Qiagen) following the manufacturer’s instructions with modifications. Temperature of stool lysis was increased from 70 (recommended temperature) to 95 °C for the lysis of bacteria that are difficult to lyse, e.g. for Gram-positive bacteria. In a later step, 400 µl supernatant (instead of the proposed volume of 200 µl) was pipetted to 15 μl of proteinase K, to which 400 µl AL buffer (instead of the proposed volume of 200 µl) was added. The spin column was loaded twice with 400 µl of the lysate. This modification resulted in a higher total DNA yield.

### 16S Ribosomal RNA Gene Sequencing

Variable (V) region 3 and 4 amplicons of 16S rRNA gene were sequenced according to the 16S metagenomic sequencing library protocol of Illumina. 16S Amplicon PCR Forward Primer 5′ TCGTCGGCAGCGTCAGATGTGTATAAGAGACAGCCTACGGGNGGCWGCAG and 16S Amplicon PCR Reverse Primer 5′ GTCTCGTGGGCTCGGAGATGTGTATAAGAGACAGGACTACHVGGGTATCTAATCC were selected as specific primers for the regions of interest of the 16S rRNA gene (V3 and V4). The final library of 96 samples was loaded on MiSeq for high-throughput sequencing (2 × 300 cycle V3 kit).

### Sequence Analysis

Quality-scored sequences were extracted from fastq files. Sequence analysis was performed using search and clustering tools from USEARCH bioinformatics platform [23–25] and different R packages.

### Calculation of Alpha Diversity

Calculation of microbial alpha diversity indices Fisher, Shannon, and Chao1 was performed using USEARCH.

(https://www.drive5.com/usearch/manual/alpha_metrics.html).

### Calculation of Relative Abundances and CVID-Specific Dysbiosis Indices

The relative abundances of taxa were calculated by dividing the number of sequences of a taxon by the total number of sequences of the respective sample. In a first step, taxa were screened for significant differences (Mann–Whitney *U* test, *p*-value < 0.05) between the study cohorts CVID and HDs.

For the heatmap of differentially abundant species the fold change of the means of the relative abundance of significantly different taxa was calculated by dividing the mean of the patient cohort by the mean of the healthy cohort. Next, CVID-specific dysbiosis indices were calculated in a similar fashion to Jørgensen et al. [8]. By dividing the sum of the relative abundances of taxa increased in CVID compared to HDs by the sum of the relative abundances of taxa decreased in CVID compared to HD, we obtained CVID-specific dysbiosis indices for every taxonomic rank in each sample.

### Measurement of Fecal Immunoglobulin Levels

The stool supernatant was collected by centrifugation of a 2 ml aliquot of stool sample. Then, Enzyme-linked Immunosorbent Assay (ELISA) with an alkaline-phosphatase system was performed. Corning 96-well half-area plates were coated with either Goat anti-human IgG-UNLB, IgA-UNLB or IgM-UNLB (Southern Biotech) in a final concentration of 5 µg/ml (total volume 30 µl). Plates were incubated overnight at 4 °C. All subsequent washing steps were performed with PBS 0.025% Tween 20 using a Microplate Washer. The plates were washed 4 times before being blocked with 75 µl PBS 10% FCS solution (blocking buffer) for 3 h. One to five dilutions of the supernatants were prepared. Standard dilution rows starting from 2 µg/ml were prepared using a human reference serum. Plates were incubated with 25 µl of the diluted supernatants and standards for 4 h at room temperature. After another washing 25 µl of the secondary antibodies diluted 1:500 in blocking buffer (Goat anti-human IgG-AP, IgA-AP and IgM-AP, Southern Biotech) were added for an incubation time of 2 h at room temperature. After washing 4 more times, 50 µl phosphatase substrate in a concentration of 1 mg/ml was added. After 30 min of incubation at 37 °C, the plates were read at 405 nm wavelength. The standard curve was calculated by the SkanIT software.

### Measurement of Fecal Protein Levels

The fecal protein concentration was measured with the Pierce BCA Protein Assay Kit (Thermo Scientific™) following the instructions of the microplate procedure suggested in the manual. One to 8 dilutions of the stool supernatant were prepared. For the standard curve, the albumin standards were diluted from 2000 μg/ml to 12.5 μg/ml. Plates were read with an ELISA reader at 562 nm wavelength. The analysis was performed with the SkanIT software.

### Normalization of Immunoglobulin Levels

The concentration of fecal immunoglobulins of each sample was normalized to the protein concentration by division. Normalization for the weight of stools was not chosen due to the variable stool composition; there was no relevant correlation between stool weight and protein concentration (Supplementary Fig. S1). In order to pool the data of two separate experiments (Exp_01 and Exp_02), the immunoglobulin concentration was normalized to the median immunoglobulin concentration of HDs of the respective experiment (Supplementary Fig. S2).

### Division of Patients into Subgroups According to Immunoglobulin Levels

Based on the distribution of immunoglobulin levels of HDs, patients were divided into further subgroups. Patients with an IgA level (normalized to the protein) below the first quartile of the levels of HDs belonged to the IgA_low group. Patients with levels above the first quartile belonged to the IgA_norm group. For IgM, we proceeded in the same way (IgM_low and IgM_norm). For IgG, we chose the third quartile as the cut-off level, because the patients had significantly higher IgG levels than HDs. Patients with levels above the third quartile of the levels of HDs belonged to the IgG_high group, patients with levels below the third quartile belonged to the IgG_norm group.

### Statistical Analysis

If not otherwise stated, significance was calculated using the Mann–Whitney *U* test. A *p*-value of < 0.05 was considered statistically significant. Q-values (adjusted *p-*values) were calculated by false discovery rate (FDR) analysis using the Storey procedure [26]. GraphPadPrism 5 was used to create the figures. The Venny program was used for Figs. 7 and S3 [27].

## Results

### Description of the Study Population

#### Epidemiological Characteristics of the Study Groups

The study population consisted of 28 CVID samples and a total of 42 HD samples, which included 21 samples from the corresponding HHDs of the patients. Their epidemiological characteristics are indicated in Table 1. The percentage of male participants was 39% in the patient and 46% in the HD cohort. The mean age at the time of sampling was not significantly higher in HDs. Three of the HDs were smokers at the time of this study, while there were only non-smokers in the patient cohort. Patients had a lower body mass index (BMI) than HDs, which was not statistically significant. The proportion of people on an omnivore diet was comparable in the study groups.

**Table 1:** Epidemiological characteristics of the study groups

	CVID	HD	*p*-value
Number	28	42	-
Sex ratio, % male	39.3	46.3	0.14
Age, mean + / − SD	48, + / − 16	54, + / − 13	0.11
Smokers *, %	0	8.1	0.08
BMI, mean + / − SD	24.7, + / − 5.8	26.5, + / − 5.2	0.06
Omnivore diet *, %	88.5	94.4	0.15

The data was collected by means of questionnaires. *P*-values for age and BMI were calculated using the Mann–Whitney *U* test. The other *p*-values were determined by the Chi-square test

*For the parameters marked with an asterisk, few study participants did not provide any information and thus were excluded from the analysis

#### Clinical Phenotype of the CVID Patient Group

All patients suffered from recurrent infections. Almost eighty percent of CVID patients additionally presented with CVID-associated non-infectious complications (Table 2), of which splenomegaly was most prevalent (50%). Other frequent complications included lymphadenopathy (28.6%), autoimmunity (39.3%), enteropathy (21.4%), and granulomatous disease (32.1%). Patients presenting with these complications are defined as the patient group with immune dysregulation (*n* = 22) as opposed to the group with infections only (*n* = 6).

**Table 2:** Clinical phenotype of the CVID patient group (*n* = 28)

Manifestation	Number	(%)
Infections only	6	(21.4)
Complications	22	(78.6)
Splenomegaly	14	(50.0)
Lymphadenopathy	8	(28.6)
Autoimmunity	11	(39.3)
Enteropathy	6	(21.4)
Granulomatous disease	9	(32.1)

The number of patients and the ratio of patients with the respective clinical manifestation are indicated. Patients with complications presented at least one of the listed conditions in addition to recurrent infections. Supplementary Table 1 shows the complete list of clinical manifestations for every patient

The data was extracted from the patients’ medical letters

Within the patient group with immune dysregulation, the occurrence of a particular complication did not correlate with the occurrence of another complication (Supplementary Fig. S3). The main laboratory data and the CVID class (Freiburg, EUROclass) of the patients are summarized in the Supplementary Tables S2.1–2.2.

Twenty-six of the 28 patients were under IgG replacement therapy, most of whom had a subcutaneous administration (*n* = 20). Regarding immunomodulatory medication other than immunoglobulin replacement, three patients took low-dose glucocorticoids. As part of the exclusion criteria, patients with an antibiotic intake within the last 4 weeks before sampling were excluded from the study population. One patient had taken an antibiotic within the last 6 weeks. Two HDs and 12 CVID patients had received antibiotics within the last 6 months.

### Alpha Diversity of Gut Microbiota in CVID

**Fig. 1:**
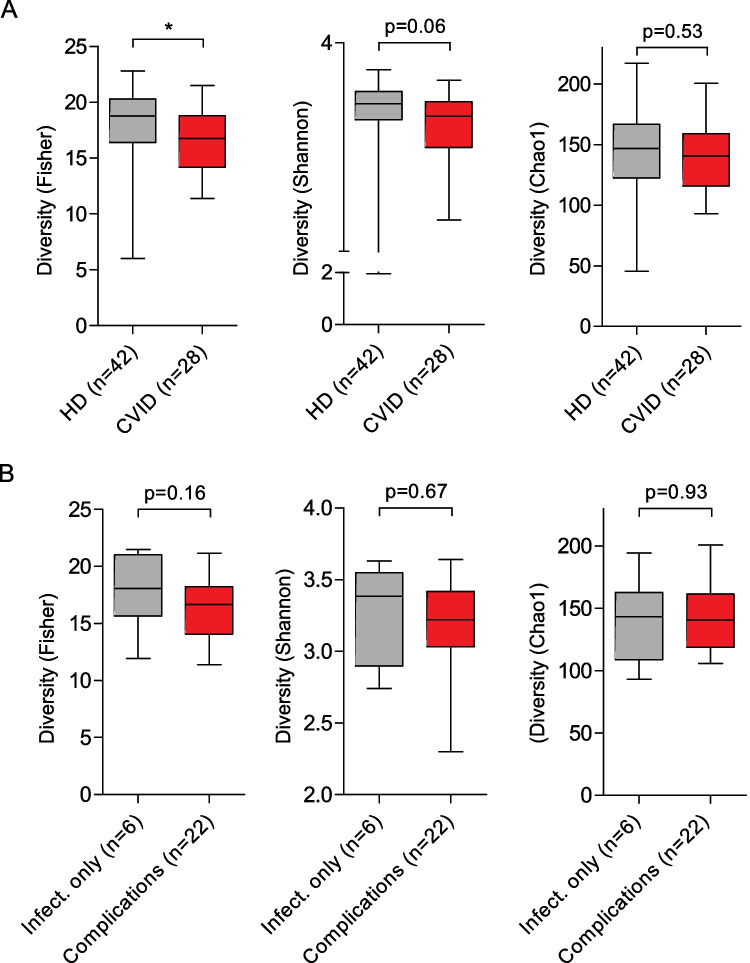
Alpha diversity of gut microbiota in CVID. Box plots displaying the microbial diversity indices Fisher, Shannon, and Chao1 for (**A**) samples of HDs (*n* = 42) and CVID patients (*n* = 28), (**B**) CVID patients with infections only (*n* = 6) and immune dysregulation (*n* = 22). The box of the box plots ranges from the 25th to 75th percentiles. The whiskers indicate the minimum and maximum values and the median is indicated by a black line within the box. Significant differences calculated with Mann–Whitney *U* test are highlighted by asterisks (*** *p* < 0.0005, ** *p* < 0.005, and * *p* < 0.05)

Microbial alpha diversity describes the diversity of species within a single sample, which can be quantified with different measures (diversity indices) [28]. Alpha diversity depends on richness (total number of species) and evenness (similarity in species relative abundances) [29]. We measured reduced Fisher (mean 16.75 vs. 17.88) and Shannon (mean 3.20 vs. 3.32) indices, estimators of species richness and evenness [30, 31], in stool samples from CVID patients compared to HDs (Fig. 1A). Of these, only the Fisher index reached statistical significance. The Chao1 index, an estimator of species richness [30], did not differ. Within the patient cohort, there was a non-significant trend towards lower microbial diversity in patients with signs of immune dysregulation as measured by Fisher indices, while the Shannon and Chao1 indices were comparable (Fig. 1B). Antibiotics intake within the last 6 months before stool sampling was not associated with decreased microbial alpha diversity (Supplementary Fig. S4).

### Altered Gut Microbiome Composition in CVID

**Fig. 2:**
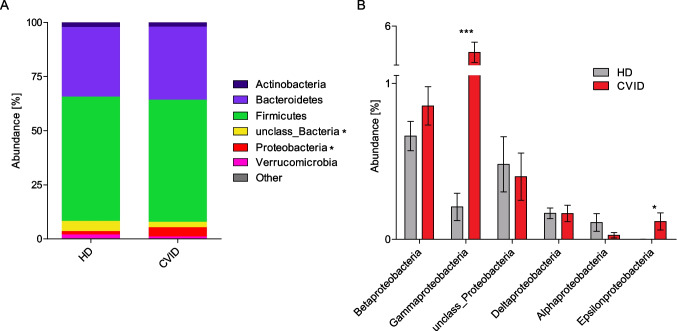
Altered gut microbiome composition in CVID. (**A**) Stacked bar diagrams showing the mean relative abundance of the indicated phyla of HD samples (*n* = 42) and CVID patients (*n* = 28). (**B**) Bar diagrams displaying the mean relative abundance and standard deviation of the indicated classes within the phylum *Proteobacteria* of HD samples (*n* = 42) and CVID patients (*n* = 28). Significant differences calculated with Mann–Whitney *U* test are highlighted by asterisks (*** *p* < 0.0005, ** *p* < 0.005, and * *p* < 0.05)

We identified a large number of differentially abundant taxa in CVID patients compared to HDs (Supplementary Tables S3.1–3.6). On the level of phylum, we detected a significantly higher relative abundance of *Proteobacteria* together with a lower proportion of unclassified bacteria in samples of CVID patients compared to HDs (Fig. 2A). The increase in *Proteobacteria* derived from an increase of the classes *Gamma*- and *Epsilonproteobacteria* (Fig. 2B).

Within the class *Gammaproteobacteria*, its order *Enterobacterales*, its family *Enterobacteriaceae*, and its genus *Escheria* were more abundant in CVID patients. The class *Epsilonproteobacteria* was enriched of its order *Campylobacterales*, its family *Campylobacteraceae*, its genus *Campylobacter*, and its species *Campylobacter concisus*.

**Fig. 3:**
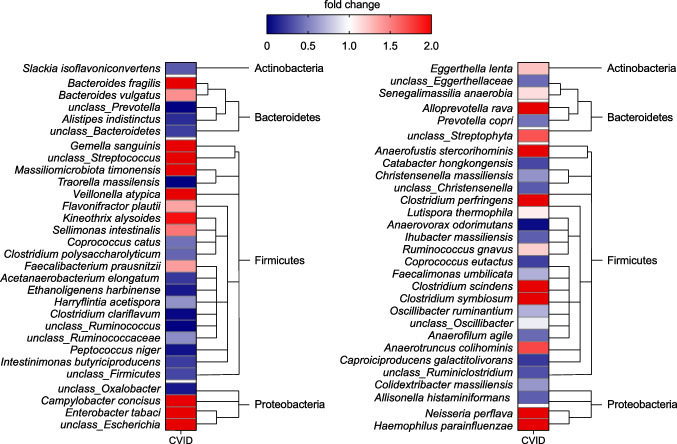
Different gut microbiome composition on the level of species. The fold changes of the relative abundances of the indicated species of CVID patients compared to HDs are shown in this dual-gradient heatmap. The significantly different species (*p*-value < 0.05, Mann–Whitney *U* test) with a calculable fold change are shown. Red color indicates species that were more abundant in CVID, whereas the blue color indicates lower abundance. Cluster trees on the right hand of the heatmaps indicate the taxonomic hierarchy. For visualization purposes, the fold change is limited to a maximum value of 2

The species that were significantly more abundant in CVID included *B. fragilis*, *Veillonella atypica*, *Neisseria perflava*, *Haemophilus parainfluenzae*, and unclassified *Escheria*. Among others, the species *Slackia isoflavoniconvertens*, *Alistipes indistinctus*, *Acetanaerobacterium elongatum*, and *Intestinimonas butyriciproducens* were less abundant in stool samples from CVID patients than in HDs (Fig. 3).

### Decreased Level of Fecal IgA in CVID

**Fig. 4:**
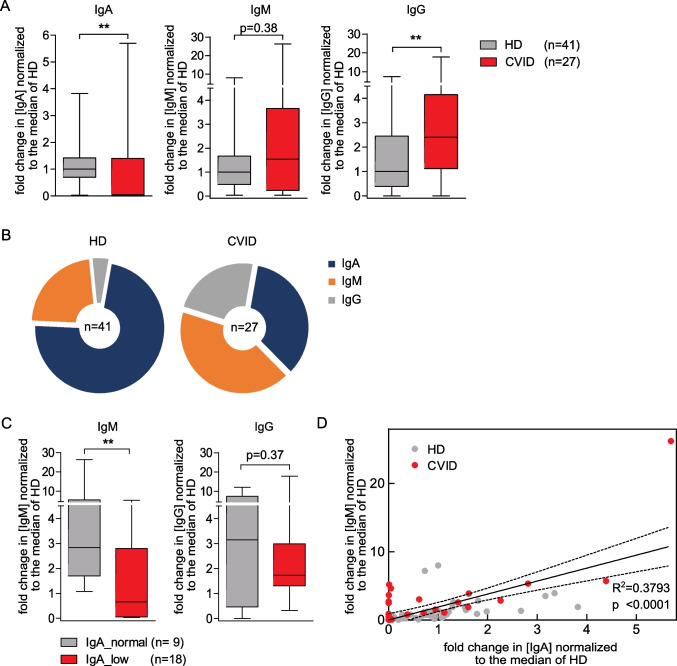
Fecal levels of IgA, IgM, and IgG. (**A**) Box plots displaying the fecal IgA, IgM, and IgG levels measured by ELISA for HDs (*n* = 41) and CVID patients (*n* = 27). The fold changes in the immunoglobulin concentration normalized to the median of HDs are shown. (**B**) Composition of fecal immunoglobulins. Means of the percentage of IgA, IgM, and IgG of total fecal immunoglobulin concentration are displayed for HDs (*n* = 41) and CVID patients (*n* = 27). (**C**) Box plots displaying the fecal IgM and IgG levels measured by ELISA for samples of CVID patients with normal (*n* = 9) and low fecal IgA levels (*n* = 18). The box of the box plots ranges from the 25th to 75th percentiles. The whiskers indicate the minimum and maximum values and the median is indicated by a black line within the box. (D) Scatter plot of the fold change in IgM over IgA in CVID patients (*n* = 27, depicted in red) and HDs (*n* = 41, depicted in grey). The common best-fit line for IgM for HDs and CVIDs and the 95% confidence interval are depicted (slope 1.899 ± 0.2991, *R*.^2^ = 0.3793, *p* < 0.0001). Significant differences calculated with Mann–Whitney *U* test are highlighted by asterisks (*** *p* < 0.0005, ** *p* < 0.005, and * *p* < 0.05)

The measurement of fecal levels of IgA, IgM, and IgG, revealed significantly lower IgA and higher IgG levels in CVID patients than in HDs, while the IgM level did not differ (Fig. 4A). Furthermore, the composition of the fecal immunoglobulins was altered (Fig. 4B). While IgA accounted for the largest proportion of fecal immunoglobulins in HDs, CVID patients had, on average, a similar proportion of IgM, IgG, and IgA.

For the further analysis, patients were divided into subgroups based on the distribution of immunoglobulin levels of the HDs (see Methods).

Fecal immunoglobulin levels did not correlate with the occurrence of CVID-associated complications (Supplementary Fig. S5), possibly due to the limited number of study participants.

In patients with selective IgA deficiency, there is evidence that the lack of fecal IgA may be counterbalanced by increased IgM [32]. Unlike in patients with selective IgA deficiency, CVID patients with low fecal IgA levels also had significantly lower fecal IgM levels than patients with normal fecal IgA levels (Fig. 4C). Importantly, we also measured a slight positive correlation between fecal IgA and IgM levels in HDs (Fig. 4D). This is not in contrast to the fact that IgM levels did not differ between HDs and CVID patients (Supplementary Fig. S6).

### Fecal IgA and IgM Levels Correlate with Microbial Diversity and Dysbiosis in CVID

**Fig. 5:**
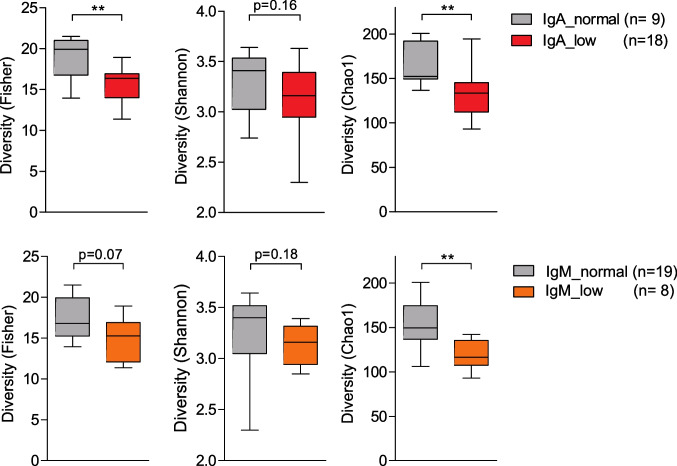
Correlation of microbial alpha diversity with fecal immunoglobulin levels. Box plots displaying the microbial alpha diversity indices Fisher, Shannon, and Chao1 for samples of CVID patients with normal (*n* = 9) and low fecal IgA levels (*n* = 18), and samples of CVID patients with normal (*n* = 19) and low fecal IgM levels (*n* = 8). The box of the box plots ranges from the 25th to 75th percentiles. The whiskers indicate the minimum and maximum values and the median is indicated by a black line within the box. Significant differences calculated with Mann–Whitney *U* test are highlighted by asterisks (*** *p* < 0.0005, ** *p* < 0.005, and * *p* < 0.05)

We measured significantly decreased alpha diversity in samples from CVID patients with low to absent fecal IgA levels compared to patients with normal fecal IgA levels. Reduced microbial diversity was additionally linked to low fecal levels of IgM (Fig. 5). Notably, we did not find any association between microbial diversity and fecal IgG levels.

**Fig. 6:**
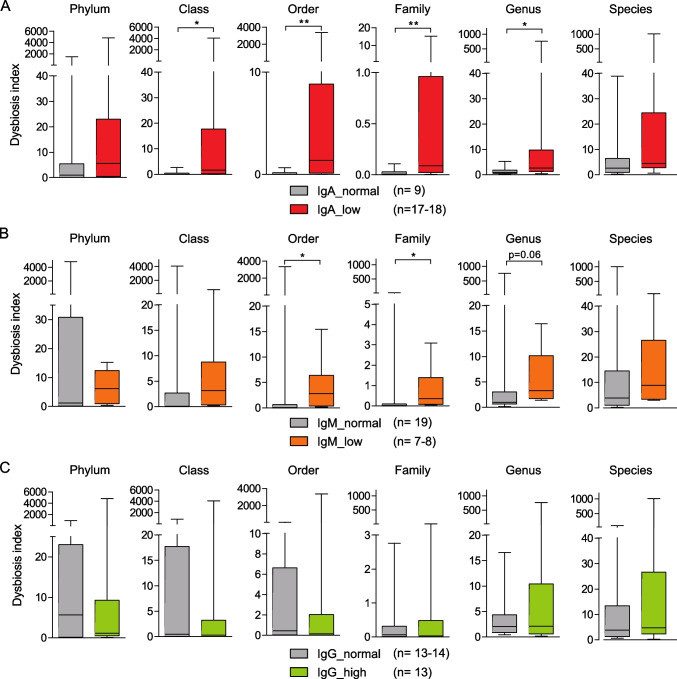
Dysbiosis is linked to low fecal levels of IgA and IgM. Box plots display the CVID-specific dysbiosis indices for phylum, order, class, family, genus, and species (from left to right) of the indicated CVID patient groups (**A**) IgA, (**B**) IgM and (**C**) IgG. Numbers in the dysbiosis index of the phylum vary, because in few cases the respective index could not be calculated. The box of the box plots ranges from the 25th to 75th percentiles. The whiskers indicate the minimum and maximum values and the median is indicated by a black line within the box. Significant differences calculated with Mann–Whitney *U* test are highlighted by asterisks (*** *p* < 0.0005, ** *p* < 0.005, and * *p* < 0.05)

Similar to Jørgensen et al., we calculated CVID-specific dysbiosis indices for each sample by considering the significantly increased and decreased taxa in CVID patients compared to HDs. We calculated six different dysbiosis indices (for every taxonomic rank). Low fecal IgA levels were associated with a higher dysbiosis index at the taxonomic ranks class, family, order, and genus, indicating that dysbiosis is also linked to low levels of fecal IgA in CVID (Fig. 6). Likewise, CVID-specific dysbiosis was more prevalent in patients with low fecal IgM levels at the taxonomic ranks order and family, while fecal IgG levels did not correlate with dysbiosis indices.

In accordance with the previous studies, CVID-specific dysbiosis was most prevalent in the patient group with CVID-associated complications at the taxonomic rank genus and species (Supplementary Fig. S7).

### IgA- and IgM-specific Taxonomic Differences in CVID

In a further analysis, we aimed to identify the significantly different taxa between samples from patients with normal and patients with decreased fecal immunoglobulin levels by comparing their relative abundance. This analysis revealed mostly an IgA-, but also an IgM-specific signature of the gut microbiome in CVID patients (Supplementary Tables S4.1–5.2).

**Fig. 7:**
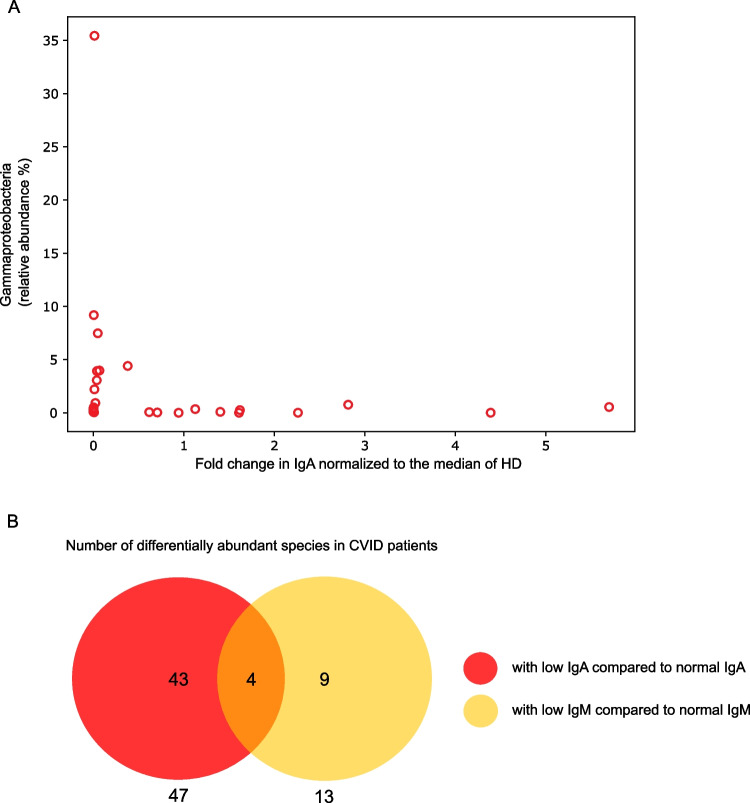
The abundance of certain taxa is dependent on fecal IgA and IgM levels. (**A**) Graph displays the fold change in fecal IgA normalized to the median of HDs and the relative abundance of *Gammaproteobacteria* (in percent) in CVID patients (*n* = 27). (**B**) Venn diagram illustrates the number of significantly different species between patients with normal and patients with reduced fecal IgA (red, *n* = 47) or IgM (yellow, *n* = 13) levels and the shared species (orange, *n* = 4) among these

Samples from CVID patients with low fecal IgA levels were significantly enriched for the class *Gammaproteobacteria* (Fig. 7A), its order *Enterobacterales*, its family Enterobacteriaceae, its genus *Escheria*, and the species unclassified *Escheria* compared to patients with normal IgA levels*.* In contrast to the limited number of increased taxa, the number of decreased taxa in samples from patients with low IgA levels was large (e.g. 3 compared to 44 at the rank of species).

Likewise, we detected a larger number of significantly decreased taxa than increased taxa in patients with low IgM levels compared to patients with normal IgM levels. Since samples from patients with low IgA or IgM levels shared the increased relative abundance of taxa belonging to the order *Enterobacterales*, we were interested in the extent of intersection of the IgA- and IgM-specific taxonomic differences. With only four shared species (Venn diagram in Fig. 7B and Supplementary Table S6), the intersection seems to be rather small, suggesting a mostly different role of IgA and IgM in the gut microbiome of CVID patients.

## Discussion

This study confirms a significantly different microbial composition of the stool in patients with CVID, when compared to HDs [8, 11]. In line with previous reports, CVID-specific dysbiosis was more prevalent in the patient group with immune dysregulation than in the patient group with infection-only CVID. Since markers for systemic immune activation such as LPS and sCD25 were shown to be especially increased in the serum of CVID patients with immune dysregulation, which correlated with decreased alpha diversity and increased dysbiosis [8], an increased translocation of gut microbiota has been proposed as driver for immune dysregulation in CVID [33].

In previous studies, reduced microbial diversity correlated with decreased serum levels of IgA in CVID patients [8, 11], suggesting that the lack of IgA contributes to the altered gut microbiome in CVID.

We measured significantly lower fecal IgA, but higher IgG levels in stool samples from CVID patients compared to HDs, while IgM levels did not differ. In contrast to selective IgA deficiency, where an increase in IgM level at the mucosa of the respiratory and gastrointestinal tract can compensate for the lack of IgA [34, 35], IgM levels were not increased in stool samples from CVID patients. Instead, the group of patients with reduced IgA levels had significantly reduced IgM levels compared to the group of patients with normal IgA levels. This positive correlation between fecal IgA and IgM levels was also discovered in HDs, but to a lesser extent.

Low fecal levels of both IgA and IgM independently correlated with increased dysbiosis and decreased microbial diversity in CVID patients. Numerous studies have demonstrated the importance of the binding of secretory IgA and IgM to microbiota for intestinal homeostasis [16–20]. The coating process induces the neutralization of microbial antigens to limit microbial stimulation, the activation of the complement system, and the agglutination of bacteria for a faster clearance [16, 36].

We here describe an IgA-, but also an IgM-specific signature of the intestinal microbiome in CVID patients. Stool samples from patients with decreased fecal IgA or IgM were not only less diverse, as shown by decreased alpha diversity, but also both, depleted and enriched for specific taxa. The increased taxa included bacteria of the class *Gammaproteobacteria* and its order *Enterobacterales.* Many taxa of the class *Gammaproteobacteria* are considered intestinal pathogens, including *Escherichia*, *Shigella*, and *Salmonella* [37]. IgA has been attributed a major role in targeting *Gammaproteobacteria* [36, 38]. The microbial composition of the gut significantly changes during lifetime. After birth, the abundance of *Proteobacteria* is the highest and decreases within the first months of life [39]. The production of *Gammaproteobacteria*-specific IgA was shown to be essential for this maturation process [39]. In IgA-deficient mice, a high abundance of *Proteobacteria* persisted, which led to an increased susceptibility to intestinal inflammation in a DSS-colitis model [39]. In a longitudinal study on the maturation of the gut in infants, Janzon et al. have shown that IgM coats a similar type of bacteria to IgA, for instance the family Enterobacteriacae belonging to the *Proteobacteria* [40]. Insufficient fecal IgA and IgM production might therefore lead to the outgrowth of these pathogens in CVID patients. In line with this, Ho et al. detected a high abundance of 16S rDNA of gut microbiota which are known to be mostly IgA-, but also IgM-coated in the serum of CVID patients [12].

In our study, fecal IgG levels were significantly increased in CVID patients compared to HDs and neither correlated with dysbiosis nor with microbial diversity. At the time of sample collection, all patients except for two were under IgG replacement therapy. This increase in IgG could be explained by an intestinal loss of the therapeutically replaced IgG via a dysfunctional gut barrier. Indeed, an elevated level of the intestinal fatty-acid binding protein (I-FABP), a protein of the epithelial gut barrier, was measured in the serum of CVID patients compared to healthy controls [12]. Importantly, this was not associated with the presence of enteropathy, indicating a common gut barrier defect in CVID [12]. Similarly, we did not find any association between the presence of enteropathy and increased IgG levels in stool.

IgG replacement therapy is effective in the protection against infections of the respiratory tract in CVID patients [7, 41]*.* Intravenous immunoglobulin replacement therapy restored CD4 T cell function and decreased levels of endotoxins in the study by Perreau et al. who suggest CD4 T cell exhaustion as a result of endotoxemia in CVID patients [42]. However, data by Fadlallah et al. questions the efficacy of IgG replacement therapy in the protection against systemic infection by gut microbiota. Patients with selective IgA deficiency were shown to compensate the lack of fecal IgA by an increased production of anti-commensal specific IgG in the serum. In CVID patients, however, serum IgG, administered through the IgG replacement therapy, only weakly bound to CVID gut microbiota, indicating the importance of an autologous generation of commensal-specific IgG to prevent infection with gut microbiota [43]. Our finding of decreased IgA and increased IgG in stool samples is in accordance with the data of a recently published study, which showed reduced IgA, increased IgG, and lower microbial diversity in the sputum of immunodeficient patients compared to healthy controls [44].

The term “dysbiosis” is increasingly but differently used in literature [45]. By comparing the gut microbiota of patients with those of a healthy study cohort, we artificially defined the most frequently altered microbiota as CVID-specific dysbiosis. Similarly, there is an ongoing debate on how to assess microbial alpha diversity [28]. There are multiple ways of calculating it with sometimes divergent results [46]. We therefore presented three different indices. Even within the healthy study population, microbiota and fecal immunoglobulin levels varied. Due to this disparity, it is important to include as many HDs and patients as possible to obtain representative data. Our study cohorts consisted of comparatively few study participants, making a subgroup analysis of patients difficult to interpret.

Collectively, we describe a mostly IgA-, but also IgM-specific signature of the gut microbiome in CVID. This highlights the important role of secretory IgA and IgM for intestinal homeostasis. In CVID, the lack of secretory IgA and IgM might contribute to the development of an altered gut microbiome and to an increased microbial translocation, possibly resulting in immune dysregulation.

## Conclusions

This study extends the knowledge about the role of gut microbiota and intestinal immunoglobulins in CVID. We confirm an altered gut microbiota composition in CVID patients compared to HDs. Correlating our microbiome data with levels of fecal immunoglobulins, we observe a link between reduced microbial diversity, dysbiosis, and decreased fecal IgA and IgM. As a key finding, we identify fecal immunoglobulins as a potential modifier of the gut microbiome in CVID patients.

## Supplementary Information

Below is the link to the electronic supplementary material.

Supplementary file1 (DOCX 1416 KB)

## Data Availability

The datasets generated during and/ or analyzed during the current study are available from the corresponding author on reasonable request.
