# Supplementary material for: Fecal Immunoglobulin Levels as a Modifier of the Gut Microbiome in Patients with Common Variable Immunodeficiency
Source: J Clin Immunol. 2023 Mar 24;43(6):1208–20. doi: 10.1007/s10875-023-01469-9 (PMC10354144; doi:10.1007/s10875-023-01469-9)
Supplement: Supplementary file 1 — Supplementary file1 (DOCX 1416 KB) [file 10875_2023_1469_MOESM1_ESM.docx]

## Supplementary Data

**Table S1** Clinical manifestations of the CVID patient cohort (n=28).

If a symptom applies to a patient, this is marked with the number 1, as opposed to 0, which means that the symptom was not detected. If one of the main categories lymphoproliferation, autoimmunity, enteropathy or granulomatous disease applies to a patient, he belongs to the group of patients with immune dysregulation. The data was extracted from the patients’ medical letters.

The acronym GLILD stands for granulomatous lymphocytic interstitial lung disease.

**Table S2.1** Clinical and laboratory data of the CVID patients 1-14.

Laboratory data is taken from the physician letters of the Center for Chronic Immunodeficiency.

For serum immunoglobulins, the levels closest to the time of stool collection were considered (time interval of a mean of 3.2 years and a median of 1 year between serum and stool collection).

The abbreviation "NA" stands for not assigned.

**Table S2.2** Clinical and laboratory data of the CVID patients 15-28.

Laboratory data is taken from the physician letters of the Center for Chronic Immunodeficiency. For serum immunoglobulins, the levels closest to the time of stool collection were considered (time interval of a mean of 3.2 years and a median of 1 year between serum and stool collection).

The abbreviation "NA" stands for not assigned.

**Table S3.1** Differential abundant taxa in CVID compared to HD on the phylogenetic level of phylum.

Significantly increased and decreased phyla are listed (*p-*value < 0.05; Mann-Whitney U test). The phylogenetic lineage, the OTU number, the mean of the relative abundance, the standard deviation (SD), the *p*-value, the *q-*value (FDR analysis) and the fold change (CVID/HD), if calculable, are shown. NA stands for not calculable (division by 0).

**Table S3.2** Differential abundant taxa in CVID compared to HD on the phylogenetic level of class.

Significantly increased and decreased classes are listed (*p-*value < 0.05; Mann-Whitney U test). The phylogenetic lineage, the OTU number, the mean of the relative abundance, the standard deviation (SD), the *p*-value, the *q-*value (FDR analysis) and the fold change (CVID/HD), if calculable, are shown. NA stands for not calculable (division by 0).

**Table S3.3** Differential abundant taxa in CVID compared to HD on the phylogenetic level of order.

Significantly increased and decreased orders are listed (*p-*value < 0.05; Mann-Whitney U test). The phylogenetic lineage, the OTU number, the mean of the relative abundance, the standard deviation (SD), the *p*-value, the *q-*value (FDR analysis) and the fold change (CVID/HD), if calculable, are shown. NA stands for not calculable (division by 0).

**Table S3.4** Differential abundant taxa in CVID compared to HD on the phylogenetic level of family.

Significantly increased and decreased families are listed (*p-*value < 0.05; Mann-Whitney U test). The phylogenetic lineage, the OTU number, the mean of the relative abundance, the standard deviation (SD), the *p*-value, the *q-*value (FDR analysis) and the fold change (CVID/HD), if calculable, are shown. NA stands for not calculable (division by 0).

**Table S3.5** Differential abundant taxa in CVID compared to HD on the phylogenetic level of genus.

Significantly increased and decreased genera are listed (*p-*value < 0.05; Mann-Whitney U test). The phylogenetic lineage, the OTU number, the mean of the relative abundance, the standard deviation (SD), the *p*-value, the *q-*value (FDR analysis) and the fold change (CVID/HD), if calculable, are shown. NA stands for not calculable (division by 0).

**Table S3.6** Differential abundant taxa in CVID compared to HD on the phylogenetic level of species.

Significantly increased and decreased species are listed (*p-*value < 0.05; Mann-Whitney U test). The phylogenetic lineage, the OTU number, the mean of the relative abundance, the standard deviation (SD), the *p*-value, the *q-*value (FDR analysis) and the fold change (CVID/HD), if calculable, are shown. NA stands for not calculable (division by 0).

**Table S4.1** Increased taxa in CVID patients with low fecal IgA level.

Significantly increased taxa in CVID patients with decreased fecal IgA level (below the first quartile of the HDs, n=18) compared to patients with normal IgA level (above the first quartile of the HDs, n=9) according to the Mann-Whitney U test. The phylogenetic level, the lineage, the OTU number, the means of the relative abundances, the standard deviations (SD), and the *p*-value (Mann-Whitney U test) are shown.

**Table S4.2** Decreased taxa in CVID patients with low fecal IgA level.

Significantly decreased taxa in CVID patients with decreased fecal IgA level (below the first quartile of the HDs, n=18) compared to patients with normal IgA level (above the first quartile of the HDs, n=9) according to the Mann-Whitney U test. The phylogenetic level, the lineage, the OTU number, the means of the relative abundances, the standard deviations (SD), and the *p*-value (Mann-Whitney U test) are shown.

**Table S5.1** Increased taxa in CVID patients with low fecal IgM level.

Significantly increased taxa in CVID patients with decreased fecal IgM level (below the first quartile of the HDs, n=8) compared to patients with normal IgM level (above the first quartile of the HDs, n=19) according to the Mann-Whitney U test. The phylogenetic level, the lineage, the OTU number, the means of the relative abundances, the standard deviations (SD), and the *p*-value (Mann-Whitney U test) are shown.

**Table S5.2** Decreased taxa in CVID patients with low fecal IgM level.

Significantly decreased taxa in CVID patients with decreased fecal IgM level (below the first quartile of the HDs, n=8) compared to patients with normal IgM level (above the first quartile of the HDs, n=19) according to the Mann-Whitney U test. The phylogenetic level, the lineage, the OTU number, the means of the relative abundances, the standard deviations (SD), and the *p*-value (Mann-Whitney U test) are shown.

**Table S6** Shared differentially abundant taxa among CVID patients with low fecal IgM or IgA level.

Intersection of significantly differentially abundant taxa in CVID patients with decreased fecal IgM or IgA level (below the first quartile of the HDs; IgA/M_low) compared to patients with normal IgM or IgA level (above the first quartile of the HDs, IgA/M_norm). The phylogenetic level, the lineage, the OTU number, the *p*-values for IgM and IgA (Mann-Whitney U test), and the cohort with the increase in the respective taxon are indicated.


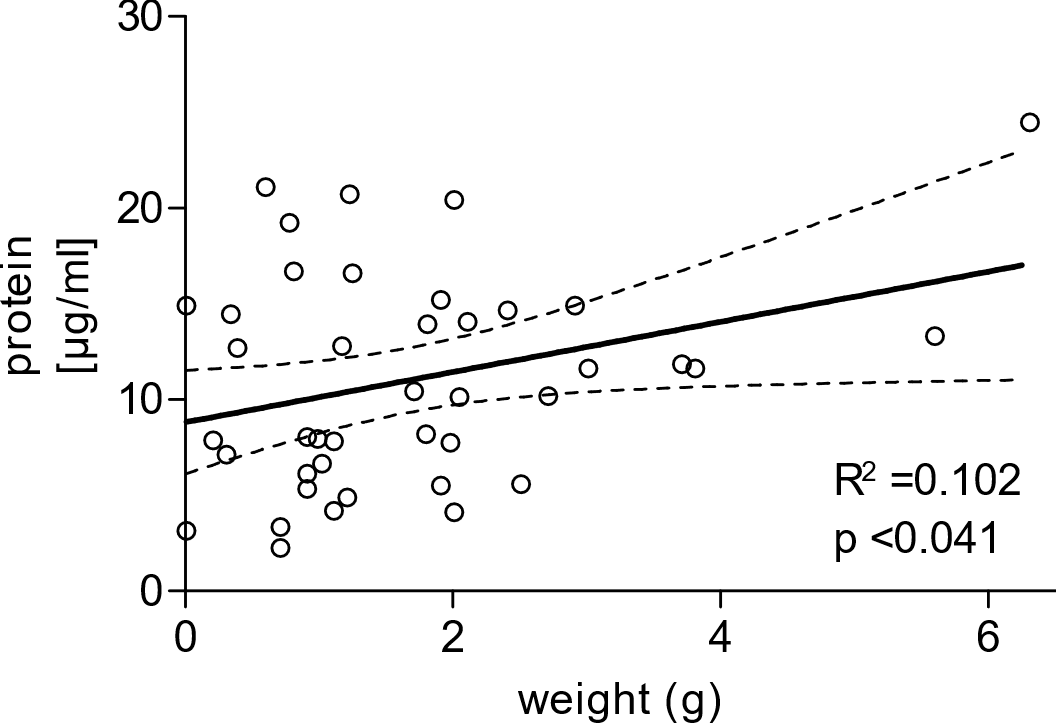


**Fig. S1** Correlation analysis between protein concentration and weight.

Scatter dot plot displays the protein concentration in µg/ml over the weight in gram for a total of 41 samples (25 samples from HDs and 16 CVID patients). The common best-fit line and the 95% confidence interval are depicted (slope 1.312 ± 0.6217, R^2^=0.1024, p= 0.0414).


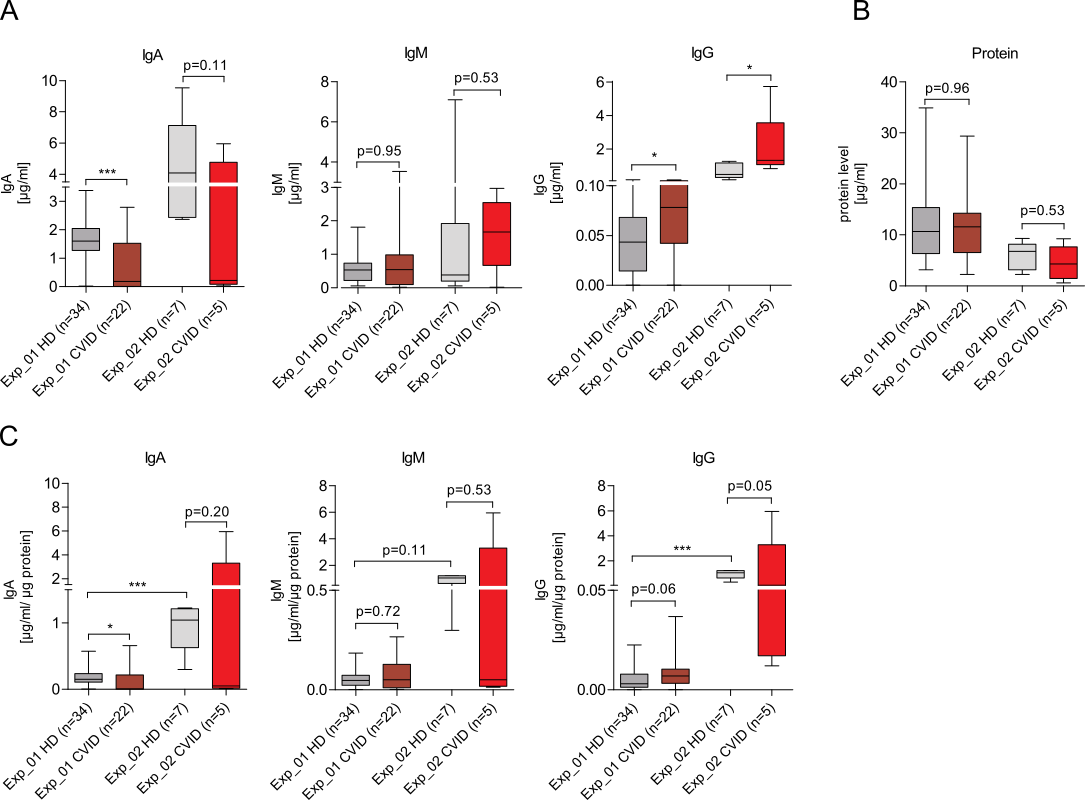


**Fig. S2** Comparison between the two experiments on fecal immunoglobulin levels.

Box plots displaying

(**A**) the concentration of IgA, IgM, and IgG in µg/ml in fecal supernatants,

(**B**) the concentration of protein in µg/ml in fecal supernatants,

(**C**) the concentration of IgA, IgM, and IgG in µg/ml per µg protein in fecal supernatants of stool samples from 34 HDs and 22 patients (Exp_01) and 7 HDs and 5 patients (Exp_02).

The box of the box plots ranges from the 25th to 75th percentiles. The whiskers indicate the minimum and maximum values and the median is indicated by a black line within the box. Significant differences calculated with Mann–Whitney U test are highlighted by asterisks (*** p<0.0005, ** p<0.005, and * p<0.05).

Despite the use of the same standard solution, significantly higher concentrations were measured for HDs in the second experiment. The differences, i.e. the qualitative statement, remained, but the quantitative values of the two experiments, despite normalization for protein, differed. Therefore, fecal immunoglobulin levels were normalized for the median of HDs included in the respective experiments.


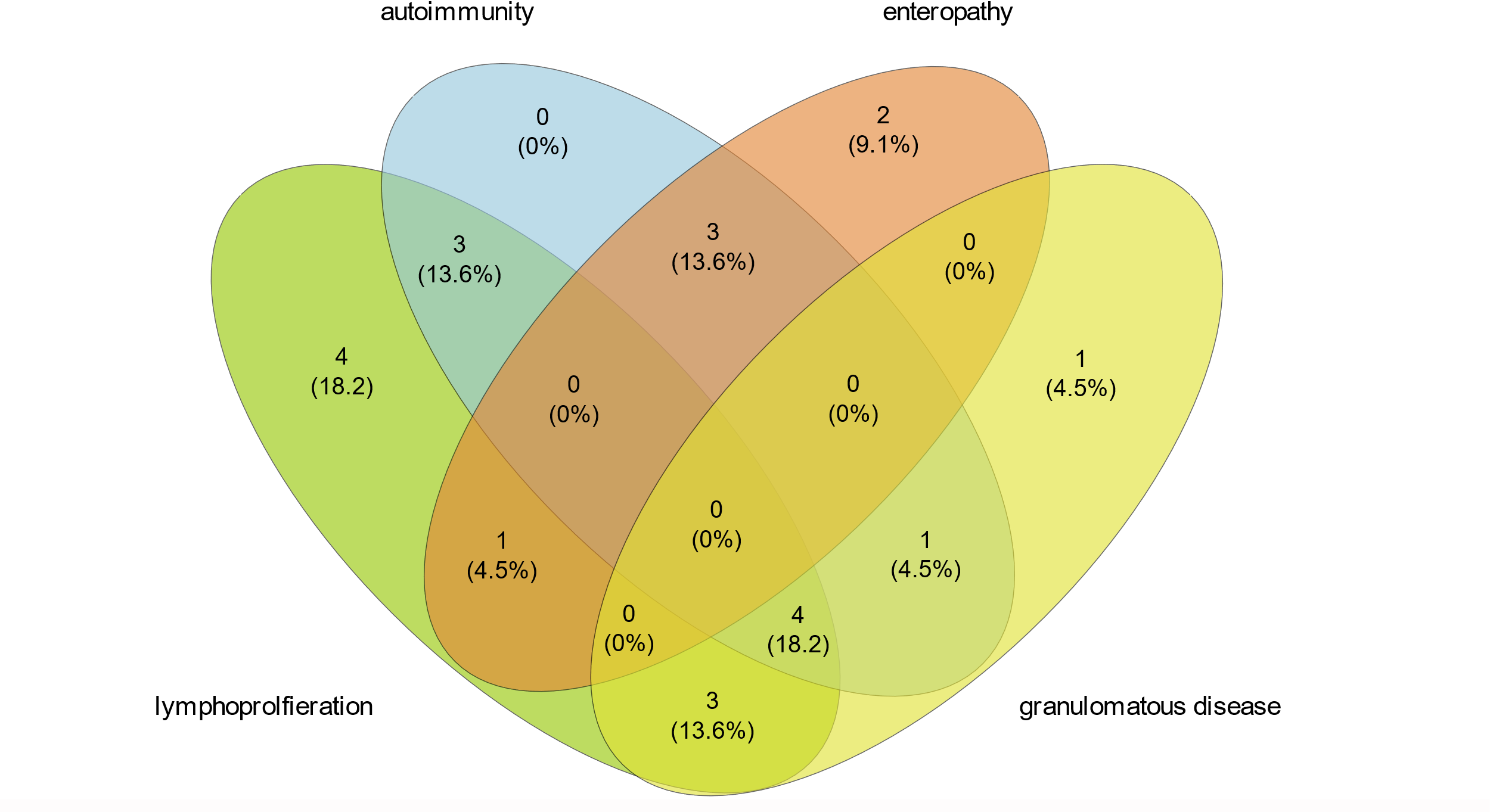


**Fig. S3** Shared clinical complications in the CVID patient group.

Venn diagram illustrates the total number of shared clinical complications lymphoproliferation (splenomegaly and/or lymphadenopathy), autoimmunity, enteropathy, and granulomatous disease within the patient group with immune dysregulation (n=22). Calculations were determined using the Venny program (by Juan Carlos Oliveros).

.

*
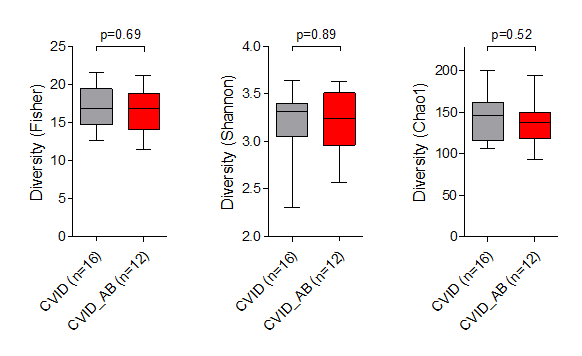
*

**Fig. S4** Diversity of gut microbiota and antibiotics intake within the last 6 months.

Box plots displaying the microbial diversity indices Fisher, Shannon, and Chao1 for CVID patients without an intake of antibiotics within the last 6 months (n=16) and patients with an intake of antibiotics within the last 6 months before stool sample collection (n=12).

The box of the box plots ranges from the 25th to 75th percentiles. The whiskers indicate the minimum and maximum values and the median is indicated by a black line within the box. Significant differences calculated with Mann-Whitney U test are highlighted by asterisks (*** p<0.0005, ** p<0.005, and * p<0.05).

**Fig. S5** Correlation of clinical manifestations with fecal immunoglobulin levels.

Box plots displaying the fold change of the indicated fecal immunoglobulin levels for specific subgroups of CVID patients:

(**A**) patients with infections only (n=5) vs. complications (n=22)

(**B**) patients without enteropathy (n=21) vs. with enteropathy (n=6)

(**C**) patients without lymphoproliferation (n=12) vs. with lymphoproliferation (n=15)

(**D**) patients without autoimmune complications (n=17) vs. with autoimmune complications (n=11)

(**E**) patients without granulomatous disease (n=19) vs. with granulomatous disease (n=9).

The box of the box plots ranges from the 25th to 75th percentiles. The whiskers indicate the minimum and maximum values and the median is indicated by a black line within the box. Significant differences calculated with Mann-Whitney U test are highlighted by asterisks (*** p<0.0005, ** p<0.005, and * p<0.05).


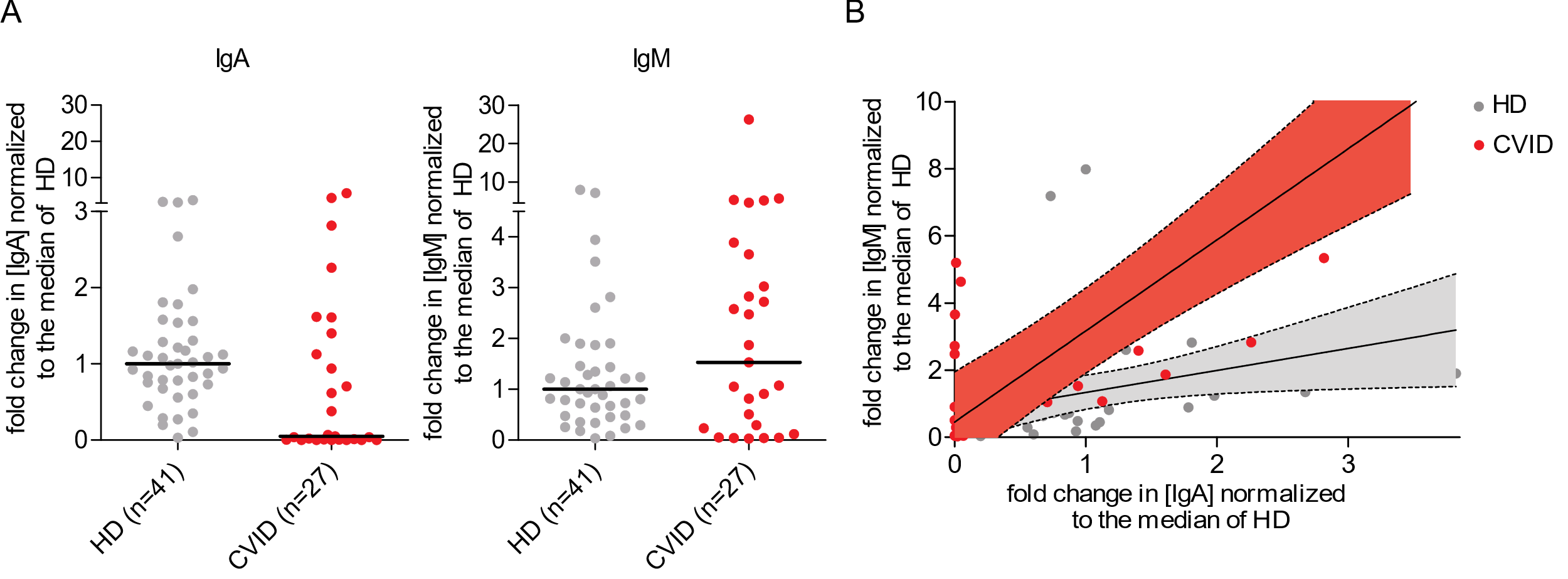


**Fig. S6** Distribution of fecal IgA and IgM within the study cohorts.

(**A**) Scatter dot plots displaying the fold changes in the fecal immunoglobulin A and M concentration normalized to the median of HDs for HDs (n=41) and CVID patients (n=27). The median is indicated by a black line.

(**B**) Scatter plot displaying the fold change in IgM over the fold change in IgA for every sample of CVID patients (n=27, depicted in red) and HDs (n=41, depicted in grey).

The common best-fit line for IgM and the 95% confidence interval are depicted in grey for HDs patients and in red for CVID patients (HD: slope 0.6567 ± 0.2982, R^2^=0.1106, p=0.0336; CVID patients: slope 2.713 ± 0.4344, R^2^= 0.6095, p< 0.0001). For visualization purposes, two data points from CVID patients are not displayed, as they are outside of the chosen axis limits.

**Fig. S7** Dysbiosis is more prevalent in CVID patients with complications.

Box plots displaying the CVID-specific dysbiosis indices for phylum, order, class, family,

genus, and species (from left to right). CVID patients with infections only (n=6)

are compared with patients with CVID-associated complications (n=22). The box of the box plots ranges from the 25th to 75th percentiles. The whiskers indicate the minimum and maximum values and the median is indicated by a black line within the box. Significant differences calculated with Mann-Whitney U test are highlighted by asterisks (*** p<0.0005, ** p<0.005, and * p<0.05).
